# First Whole-Genome Sequence and Flow Cytometry Genome Size Data for the Lichen-Forming Fungus *Ramalina farinacea* (Ascomycota)

**DOI:** 10.1093/gbe/evad074

**Published:** 2023-05-07

**Authors:** Theo Llewellyn, Sahr Mian, Rowena Hill, Ilia J Leitch, Ester Gaya

**Affiliations:** Comparative Fungal Biology, Royal Botanic Gardens, Kew, Richmond, United Kingdom; Department of Life Sciences, Imperial College London, Ascot, Berkshire, United Kingdom; Science and Solutions for a Changing Planet Doctoral Training Partnership, Grantham Institute, Imperial College London, South Kensington, London, United Kingdom; Comparative Fungal Biology, Royal Botanic Gardens, Kew, Richmond, United Kingdom; Comparative Fungal Biology, Royal Botanic Gardens, Kew, Richmond, United Kingdom; School of Biological and Behavioural Sciences, Queen Mary University of London, London, United Kingdom; Comparative Fungal Biology, Royal Botanic Gardens, Kew, Richmond, United Kingdom; Comparative Fungal Biology, Royal Botanic Gardens, Kew, Richmond, United Kingdom

**Keywords:** genome size, lichen, long-read sequencing, metagenomics, Ramalinaceae, symbiosis

## Abstract

Lichen-forming fungi are a diverse and ecologically important group of obligate mutualistic symbionts. Due to difficulties with maintaining them in culture and their extremely slow growth, lichenologists are increasingly opting for metagenomic sequencing followed by symbiont genome separation using bioinformatic pipelines. However, without knowing the true genome size of the lichen-forming fungus, we cannot quantify the completeness of the genome assembly and the efficacy of the bioinformatic filtering. To address this issue, we report here the first whole-genome assembly for the lichen-forming fungus *Ramalina farinacea* (L.) Ach. sequenced with Oxford Nanopore long-read technology alongside direct measurements of its genome size using flow cytometry. The assembly showed high contiguity (N50 = 1.55 Mb) and gene set completeness (BUSCO = 95.8%). The highly robust genome size of 33.61 Mb/1C (coefficients of variation = 2.98) that was obtained showed our assembly covered 97% of the entire genome. Our results demonstrate that accurate genome size measurements can be obtained directly from lichen thalli and used to provide a benchmark for assessing true cytometric completeness of metagenome-derived assemblies.

**Significance**

Whole-genome sequencing of obligate fungal symbionts is increasingly possible due to advances in sequencing technology and metagenomics methods. Metagenomic pipelines have taxon filtering steps to remove nontarget organism sequences. However, these steps can be inaccurate as they rely on indirect genome assembly metrics to determine genome completeness and contamination, which are often underestimates. Here, we sequence a lichen-forming fungal symbiont genome using long-read sequencing and concurrently measure for the first time its genome size directly using flow cytometry to ensure assembly completeness and validate metagenomic filtering. This study provides a roadmap for incorporating cytometric analysis into genome-sequencing projects of lichen-forming fungi, an approach we believe is necessary for producing high-quality, uncontaminated genomes from metagenomic data.

## Introduction

Fungal symbionts make up a significant proportion of the kingdom *Fungi*, with symbiotic lifestyles such as lichens, mycorrhizae, and entomopathogens spanning all major lineages (Peay et al. 2016). Given their prevalence, sequencing the genomes of these fungi is a key step in understanding the evolution of symbiotic lifestyles (Miyauchi et al. 2020). One such group is lichen-forming fungi, of which approximately 19,000 species have been described (Lücking et al. 2017). Most lichen-forming fungi (mycobionts) cannot be cultured axenically, which makes standard laboratory practices for whole-genome sequencing challenging. As a result, metagenomic sequencing is increasingly used as an alternative in lichen genomics studies (e.g., McKenzie et al. 2020; Tzovaras et al. 2020; Allen et al. 2021; Tagirdzhanova et al. 2021). When assembling fungal symbiont genomes from metagenomic data, metrics such as assembly size and BUSCO scores are used as benchmarks for assessing assembly completeness and to ensure that sequences from the other symbionts have been removed. However, bioinformatic genome size estimates from assembly size or k-mer-based approaches can differ significantly from direct cytometric genome size measurements. This disparity can be due to the fact that BUSCOs measure only gene set completeness and cannot quantify the completeness of noncoding (repeat) regions (Le Cam et al. 2019; Hill et al. 2021). As a result, seemingly complete assemblies may be missing significant portions of the nongenic regions of the genome.

**Fig. 1.**
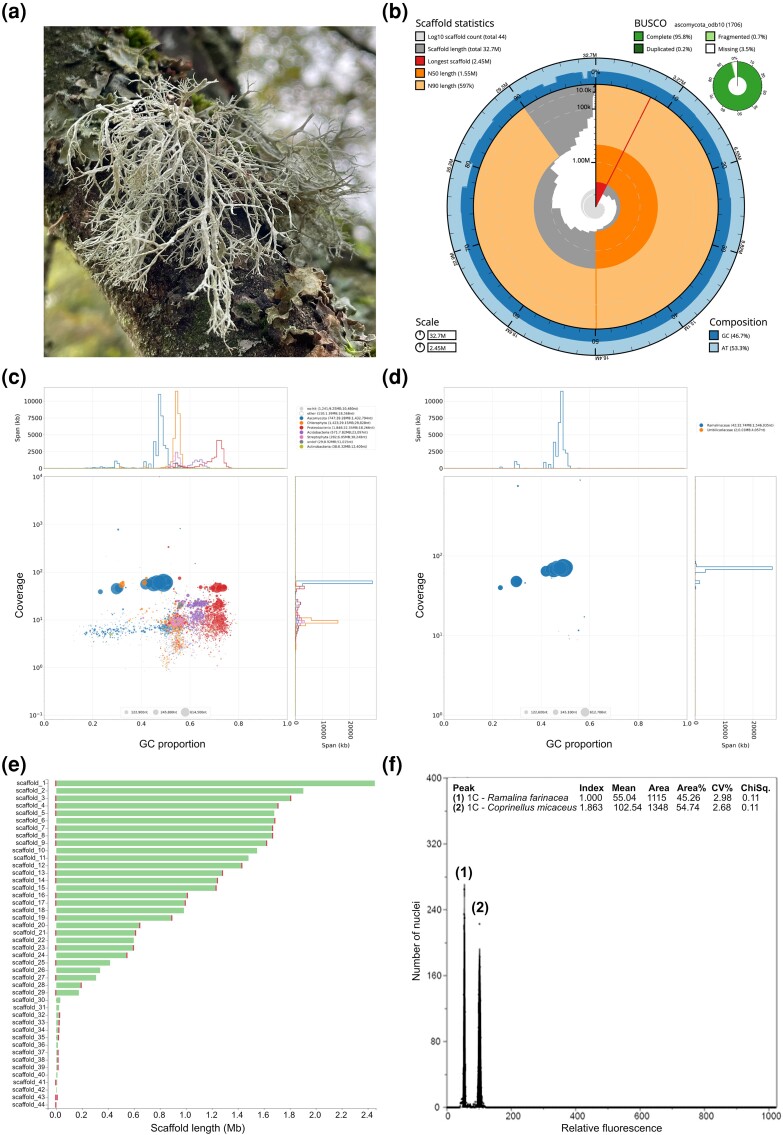
Genome assembly of *Ramalina farinacea*. (*a*) Thallus of *R. farinacea* growing on the *Quercus brantii* tree. (*b*) BlobToolKit Snailplot of mycobiont assembly showing contig length, N50/90, and BUSCO scores. (*c*) Metagenome blob plot before mycobiont filtering displaying GC-content against sequencing coverage for every contig in the metagenome assembly, with corresponding axis histograms. Color represents taxonomic identification via DIAMOND and BLAST+ searches. Circle diameter = contig length. Legend shows contig number, span, and N50 for each taxon. (*d*) Blob plot showing GC content versus coverage for isolated mycobiont genome after removing nonmycobiont contigs and reassembling (*e*) Plot showing the number and length of all 44 assembled scaffolds for the mycobiont assembly. Vertical red bars show the presence of telomere sequences (*f*) Example of a flow cytometry histogram used to estimate the genome size of *R. farinacea*. Peaks 1 and 2 correspond to propidium iodide–stained nuclei of *R. farinacea* and *Coprinellus micaceus* (internal calibration standard), respectively.

So far, no lichen-genome-sequencing studies have incorporated cytogenetic estimates of genome size made using flow cytometry. Consequently, we do not know yet how well current sequencing and assembly pipelines are performing for lichen metagenomic data. To address this issue, we concurrently performed, for the first time, long-read genome sequencing and flow cytometry genome size analysis for the lichen-forming fungus *Ramalina farinacea* (fig. 1*a*), a widespread fruticose (bushy) species in the family *Ramalinaceae* (*Lecanorales*, *Ascomycota*). We chose this species given its abundance, large thalli, and lack of a whole-genome sequence. *Ramalina farinacea* is a globally distributed epiphytic, lichen-forming fungus. It associates with a high diversity of algal symbionts predominantly from the genus *Trebouxia* (Molins et al. 2021). It is also chemically diverse, being able to produce various depside and depsidone secondary metabolites (Stocker-Wörgötter et al. 2004). Only four other *Ramalinaceae* genomes have been sequenced to date; two *Ramalina* species using short-read Illumina sequencing, and two *Bacidia* species using Oxford Nanopore long-read sequencing (Allen et al. 2021; Gerasimova et al. 2022).

## Results and Discussion

Here, we present the genome sequence for the lichenized fungus *R. farinacea* (fig. 1*b*). This is the first published whole-genome sequence for this species and the first long-read assembly for the genus. The taxonomic identification of the sample was confirmed with a blast search of the genome-derived full ITS sequence against the UNITE database (Nilsson et al. 2019) and T-BAS phylogenetic placement (Carbone et al. 2017). Both approaches confirmed the sample to be the target species *R*. *farinacea* (see Supplementary material online). Our chosen metagenomic pipeline (Llewellyn et al. 2023) successfully isolated the mycobiont reads from the metagenomic data set (fig. 1*c* and *d*). K-mer-based profiling of unprocessed mycobiont reads estimated the genome size at 31.53 Mb. The mycobiont sequence data were then assembled into 44 contigs with an assembly size of 32.74 Mb, of which 29 contigs constituted 99% (32.56 Mb) of the assembly. This falls within the range of published assembly sizes for species belonging to *Lecanoromycetes*. The assembly size was approximately 5 Mb larger than the two Illumina published *Ramalina* genomes and comparable with the *Bacidia* long-read genomes (table 1). The assembly was highly contiguous (N50 = 1.55 Mb) and the longest scaffold measured 2.45 Mb. This is at least an order of magnitude higher than the contiguity scores for the other two published *Ramalina* assemblies, confirming the trend that long-read approaches significantly improve mycobiont assembly quality. N50 is comparable with the available long-read assemblies for the *Lecanoromycetes* genera *Bacidia*, *Physcia*, *Letharia*, and *Umbilicaria* (McKenzie et al. 2020; Tzovaras et al. 2020; Wilken et al. 2020; Allen et al. 2021; Singh et al. 2021; Gerasimova et al. 2022). The assembly had a BUSCO completeness score of 95.8% (table 1).

**Table 1:** Genome Assembly Statistics for *Ramalina Farinacea* (new to This Study) Compared With Published Ramalinaceae Genome Assemblies

	Assembly Size (Mb)	N50 (Mb)	No. Scaffolds/Contigs (Mb)	BUSCO Completeness (%)	Predicted Proteins	BGCs	Bioproject Accession
*Ramalina farinacea*	32.74	1.55	44	95.8 (95.6)	8,765	52	PRJNA890456
*Ramalina intermedia*	26.25	0.273	198	95.8 (95.8)	7,355	54	PRJNA416400
*Ramalina peruviana*	26.99	0.04	1657	89.6 (89)	6,756	43	PRJNA358650
*Bacidia gigantensis*	33.12	1.81	24	93.3 (93.2)	8,400	35	PRJNA748063
*Bacidia rubella*	33.52	1.77	51	82.7 (82.6)	8,469	31	PRJNA821848

Note.—The BUSCO completeness percentages for assembled genomes from other studies were recalculated in this study to ensure comparability. Scores in brackets are single-copy BUSCOs. The BUSCO score for *Bacidia rubella* could be calculated only on the published protein set as the assembled genome has not been made publicly available. BGCs, secondary metabolite biosynthetic gene clusters.

Of the 41 contigs over 5,000 base pairs long, 13 had telomere sequences at both ends possibly implying a contig length covering a full chromosome (fig. 1*e*); a further 20 contigs had telomere sequences at one end. This result suggests the *R. farinacea* genome produced here has at least 13 chromosomes, although karyotyping would be needed to confirm this.

Genome annotation predicted 8,765 proteins, and secondary metabolite biosynthetic gene cluster (BGC) analyses identified 52 BGCs; 25 polyketide synthases (PKSs), 16 nonribosomal peptide synthetases (NRPSs), 4 terpenes, 5 NRPS-PKS hybrids, and 1 indole cluster. *Ramalina farinacea* has a similar number of BGCs to *Ramalina intermedia* and *Ramalina peruviana* which have 54 and 43 clusters, respectively (table 1). The number of BGCs is significantly higher than in the two published *Bacidia* genomes, supporting a previous observation by Gerasimova et al. (2022). The aforementioned authors attribute this to the more diverse secondary chemistry profiles of *Ramalina* species which frequently produce multiple depside and depsidones, while only a single secondary metabolite has been observed per species in the two *Bacidia* species sequenced thus far. Our new data contributes to a growing *Ramalinaceae* data set that may help inform evolutionary trends in the family. For example, to understand why *Ramalina* genomes have more diverse BGC repertoires than *Bacidia*, and whether the ancestral state of the family is a diverse BGC repertoire which was then reduced in *Bacidia* or alternatively, an ancestral BGC-poor repertoire which then expanded in *Ramalina*.

The flow cytometry histogram produced by analyzing the combined sample of propidium iodide–stained nuclei from *R. farinacea* and the internal calibration standard *Coprinellus micaceus* showed two distinct peaks (fig. 1*f*). Peak 1 (mean relative fluorescence of 55.04) corresponds to nuclei from *R. farinacea*, whereas Peak 2 (mean relative fluorescence of 102.54) comprises nuclei from *C. micaceus*. Based on the following formula: (Mean relative fluorescence of species of interest [i.e., *R. farinacea*]/Mean relative fluorescence of calibration standard [i.e., *C. micaceus*]) × 1C-value of calibration standard (i.e., 62.62 Mb), the genome size of *R. farinacea* is estimated to be 33.61 Mb. Given the low coefficients of variation of both peaks in the flow histogram (i.e., 2.98% for the *R. farinacea* [peak 1], and 2.68% for *C. micaceus* [peak 2]), the genome size estimate is considered robust, thus the assembled genome is estimated to cover 97% of the whole genome. A singe high 1C fluorescence peak for the *R. farinacea* sample suggests this individual was haploid.

Given that previously reported genome sizes for *Trebouxia* photobiont genomes estimated from genome sequencing assemblies are at least double the size of lichen mycobiont genomes (i.e., ca. 53–69 Mb; Beck et al. 2015; Kono et al. 2017; Tzovaras et al. 2020), and that no other peaks were detected in the flow cytometry analysis, we are confident that we were able to successfully separate the mycobiont tissue from the photobiont and that peak 1 in the flow histogram belongs to the mycobiont. These results demonstrate that mycobiont genome sizes can be accurately measured directly from lichen thalli, and that axenic culturing should no longer be a barrier for genome sizing of lichen mycobionts. This greatly improves the outlook for applying flow cytometry to lichen-genome-sequencing projects and supports previous work demonstrating the feasibility of flow cytometry for culture-recalcitrant fungal symbionts (Tavares et al. 2014). Our results also demonstrate that mycobiont genome size estimates from assembly size and k-mer profiles underestimate true genome size as measured by flow cytometry, a pattern recently observed in a Coleoptera data set (Pflug et al. 2020) and discussed by Hill et al. (2021) for fungi. Given the complex microbial communities found within lichen thalli and the risks associated with estimating assembly completeness using indirect metrics from assembly size, k-mer profiles, or BUSCO scores, we believe direct genome size measurement should be an essential part of future lichen metagenome genome sequencing projects.

## Materials and Methods

### Sample Collection

A fresh sample of *R. farinacea* was collected from a *Crataegus monogyna* subsp. *azarella* tree growing in the Royal Botanic Gardens Kew, UK. The thallus was cleaned of any substrate debris and 400 mg was weighed and transferred to a sterile 50 ml falcon tube. The sample was flash frozen in liquid nitrogen and ground to a fine powder using a chilled, sterile pestle and mortar. Ground material was transferred back to the falcon and immediately placed in a −80 °C freezer.

### Flow Cytometry

*Ramalina farinacea* thalli are too small to use the same individual for both DNA extraction and flow cytometry. Therefore, further samples were collected from the same host tree at the same locality for flow cytometry. Nuclear DNA content was estimated by propidium iodide flow cytometry, and *C. micaceus* was used as the calibration standard using material from a single individual that had been isolated and cultured from a collection made by R. Wright 10/05/2020 at Royal Botanic Gardens Kew, UK (culture code: FTOL_0141). The genome size of *C. micaceus* was estimated by co-running a sample with *Arabidopsis thaliana* (L.) Heynh., 1842 (ecotype col-0 NASC) with an estimated genome size of 1C = 172.44 Mb. This was achieved by using a new razor blade to co-chop a small amount of *C. micaceus* mycelium with 1 cm^2^ fresh *A. thaliana* leaf tissue in a petri dish containing 1 ml of LB01 buffer (Doležel et al. 1989). A further 1 ml of LB01 was added to the sample and the contents gently mixed. The sample was then passed through a 30-μm nylon filter, stained with 100 μl propidium iodide (1 mg/ml) and incubated on ice for 15 min. Three replicates of the combined sample were run, recording up to 1,000 nuclei per fluorescence peak using a Sysmex CyFlow Space (Sysmex Europe GmbH, Norderstedt, Germany) flow cytometer fitted with a 100-mW green solid-state laser. The resulting histograms were analyzed with the Windows™-based FlowMax software (v. 2.9 2014; Sysmex Europe GmbH), and the average of each sample was used to estimate genome size (Pellicer et al. 2021).

Once the genome size of *C. micaceus* was estimated (1C = 62.62 Mb), it was then used as the internal calibration standard for estimating the genome size of *R. farinacea*, implementing the same method as above. Initial testing showed *C. micaceus* mycelium was rich in nuclei and therefore only one forceps pinch-worth was co-chopped with approximately 0.5 cm^2^ of fresh *R. farinacea* thallus material.

### DNA Extraction and Sequencing

DNA was extracted following the steps of a modified sodium dodecyl sulfate (SDS) extraction method (Merges et al. 2021), replacing SDS with cetyltrimethyl ammonium bromide (CTAB) buffer. See Supplementary material online for a full DNA extraction protocol. The genomic DNA was eluted in 1 ml of TE buffer and column cleaned twice using QIAGEN mini-spin columns, before eluting into 50 μl of AE buffer from the Qiagen DNeasy® Plant Mini Kit (Qiagen, Redwood City, CA, USA). Extraction quality was checked using a Quantas™ Fluorometer (Promega, UK). DNA was sheared using a COVARIS-G tube, centrifuging at 4,200 rpm (1630 g) for 1 min to increase DNA throughput. The sheared DNA was converted into Oxford Nanopore libraries using the SQK-LSK110 library kit and the short fragment buffer (Oxford Nanopore Technologies, UK). Library prep followed the standard manufacturer's protocol apart from the final elution, which was done for 30 min at 37 °C. The libraries were sequenced using a SpotON R9.4.1 FLO-MIN106 flowcell for 48 h.

### Basecalling and Metagenome Assembly

Raw sequences were basecalled using the super high accuracy model (SUP) of the GPU version of Guppy v6.0.1 (config file: dna_r9.4.1_450bps_sup.cfg; Oxford Nanopore Technologies Inc.). Basecalled reads were assembled into metagenome contigs using Flye v.2.9 with the “–meta” and “–nano-hq” options (Kolmogorov et al. 2019). The *k*-mer profile of unassembled mycobiont reads was computed using Jellyfish v2.2.6 (*k* = 21) (Marçais and Kingsford 2011). The resulting *k*-mer histogram was used as input for GenomeScope to estimate genome size (Vurture et al. 2017).

### Mycobiont Filtering

Mycobiont reads were filtered from the metagenome assembly using the pipeline of Llewellyn et al. (2023). Briefly, the pipeline uses a BlobTools-based workflow (Laetsch and Blaxter 2017) and consists of DIAMOND (Buchfink et al. 2014) and BLAST+ blasts (Camacho et al. 2009), followed by reassembly of the filtered mycobiont reads. A detailed outline of all steps in each round of filtering can be found in the Supplementary material online.

Terminal telomere sequences were identified by searching for the conserved fungal telomere sequence (CCCTAA/TTAGGG) using Tapestry v1.0. (https://github.com/johnomics/tapestry). Mycobiont genome assembly contiguity was assessed using QUAST v5.0.2 (Gurevich et al. 2013) and completeness assessed using BUSCO v4.0.2 (Manni et al. 2021) and the BUSCO *Ascomycota* lineage data set (ascomycota_odb10).

To confirm that the mycobiont assembly belonged to *R. farinacea*, we extracted the universal fungal barcode ITS sequence (ITS1, 5.8S, and ITS2) using ITSx (Bengtsson-Palme et al. 2013). ITS was blasted against the UNITE database to confirm its identity (Nilsson et al. 2019). We also used blastn to extract nucLSU, mitSSU, RPB1, and RPB2 genes with GenBank sequences JQ301589, KJ766480, KJ766831, and KJ766963, respectively, as queries. These four genes were used for phylogenetic placement using the Evolutionary Placement Algorithm as implemented in T-BAS v2.3 with the Lecanoromycetes v2 reference tree (see Supplementary Figure S1) (Miadlikowska et al. 2006; Carbone et al. 2017, 2019).

### Annotation

A de novo repeat library was generated using RepeatModeler v2.0.1 with LTR discovery (Flynn et al. 2020) and used for softmasking with RepeatMasker v4.1.0 (Smit et al. 2015). Funnanotate was used to predict gene models (Palmer and Stajich 2019), incorporating expressed sequence tags and protein models from the JGI Mycocosm genomes of *Cladonia grayii*, *Usnea florida*, and *Xanthoria parietina* as evidence for EvidenceModeler (Haas et al. 2008).

Functional annotation of the predicted gene models was performed using InterProScan v5.57-90.0 (Jones et al. 2014); eggNOG-mapper v2.1.9-4dfcbd5 (Cantalapiedra et al. 2021) with sequences searched against the eggNOG orthology database v5.0.2 (Huerta-Cepas et al. 2019) using DIAMOND v2.0.15 (Buchfink et al. 2014); and antiSMASH v6.1.1 (Blin et al. 2021).

## Supplementary Material

evad074_Supplementary_Data

## Data Availability

WGS data and the annotated genome assembly are available on GenBank under the BioProject accession PRJNA890456. All bioinformatics scripts are available at https://github.com/theo-llewellyn/longread-lichens.
