# Supplementary material for: First Whole-Genome Sequence and Flow Cytometry Genome Size Data for the Lichen-Forming Fungus Ramalina farinacea (Ascomycota)
Source: Genome Biol Evol. 2023 May 7;15(5):evad074. doi: 10.1093/gbe/evad074 (PMC10195087; doi:10.1093/gbe/evad074)
Supplement: evad074_Supplementary_Data [file evad074_supplementary_data.zip › Supplementary_Material.docx]

**Supplementary Material**

First Whole Genome Sequence and Flow Cytometry Genome Size Data for the Lichen-Forming Fungus *Ramalina farinacea* (Ascomycota)

Theo Llewellyn, Sahr Mian, Rowena Hill, Ilia J. Leitch, Ester Gaya

**Materials and Methods**

*DNA extraction*

After flash freezing and grinding, the sample was transferred to a 50 ml Falcon tube with 10 ml CTAB lysis buffer (12 ml CTAB:48 μl β-mercaptoethanol) and incubated for 15 minutes at 65°C. 50 μl of RNAse A (20mg/ml) was added and the tube was incubated for 60 mins at 65°C. 10 ml of chloroform:isoamyl alcohol (24:1, v/v) was added and mixed by inversion for 5 mins. The tube was then spun at 4000 x g for 25 mins at 4°C. The upper aqueous layer was pipetted into a new Falcon tube and 0.3 volumes of 5 M potassium acetate were added. The tube was incubated for 5 mins on ice and centrifuged at 4000 x g for 20 mins at 4°C. The supernatant was transferred to a new 50 ml tube and further chloroform:isoamyl alcohol extractions were performed until no interphase between the organic and aqueous phase could be detected. Genomic DNA (gDNA) was precipitated by adding 0.1 volumes of 3 M sodium acetate (pH 5.2) and 2.5 volumes of ethanol. The sample was incubated overnight at -20°C. gDNA was centrifuged at 4000 x g for 90-180 mins at 4°C. Supernatant was discarded and the pellet was washed with 5 ml 70 % ethanol. Sample was centrifuged for 5 mins at 4500 rpm and the supernatant was poured off. The ethanol wash was repeated and any residual ethanol was removed with a pipette. The pellet was air-dryed for 5 mins and resuspended in 1 ml of 50°C TE buffer.

*Mycobiont filtering*

Mycobiont reads were filtered from the metagenome assembly using the BlobTools v1.1.1 workflow (Laetsch & Blaxter 2017). Basecalled reads were mapped to metagenome contigs using minimap2 v2.22 (Li 2018) and converted to a .bam file using samtools v1.9 (Li et al. 2009). Contigs were then taxonomically identified through a DIAMOND BLASTx (Buchfink et al. 2014) search blasted against the Uniref90 database and a BLAST+ BLASTn (Camacho et al. 2009) against a custom database of *Lecanoromycetes* class genomes downloaded from NCBI and JGI Mycocosm (both with an e-value cut-off of 1x10-25) (Grigoriev et al. 2014). Blast results were incorporated into the BlobTools workflow to visualise GC-content, coverage and taxonomic identity for each contig. Reads mapping to contigs with a top blast of either Ascomycota or ‘undefined’ that were above 20x coverage and over 5000bp long were extracted using a custom bash script.

Reads mapping to contigs with a top blast of either *Ascomycota* or ‘undefined’ that were above 20X coverage and over 5000bp long were extracted using a custom bash script. Mycobiont reads were then reassembled with Flye using the same settings as the metagenome assembly, except for also providing the genome size estimated via flow cytometry using the ‘-g’ option. The mycobiont assembly was then run through a second round of the BlobTools workflow and any remaining non-mycobiont contigs or low quality contigs (<20X, <5000 bp) were removed by a custom perl command and SeqKit’s grep function (Shen et al. 2016). We removed redundant contigs from the mycobiont assembly using redundans v0.14 (Pryszcz & Gabaldón 2016).

The remaining mycobiont contigs were error-corrected using three rounds of RACON v1.4.22 (Vaser et al. 2017) and one round of medaka v1.4.4 polishing (https://github.com/nanoporetech/medaka). The contigs were then subjected to a third and final round of the mycobiont filtering pipeline, this time retaining only contigs with a top blast for *Ramalinaceae* or any other lichen family in the *Lecanoromycetes* and incorporating an additional blastn search against the NCBI nucleotide (nt) database. All bioinformatics scripts are available at <https://github.com/theo-llewellyn/longread-lichens>.

**Results**

UNITE taxonomic identification showed a top hit to *Ramalina farinacea* with an E-value of 0.0, a UNITE score of 805.0 and 99.58% identity. T-BAS analysis, of the combined four loci dataset (nucLSU, mitSSU, RPB1 and RPB2) placed our sequences as sister to *Ramalina farinacea* with a likelihood weight of 1.0 (Supplementary Figure S1). All data related to T-BAS and UNITE analyses have been uploaded to Zenodo repository doi:10.5281/zenodo.7682356.


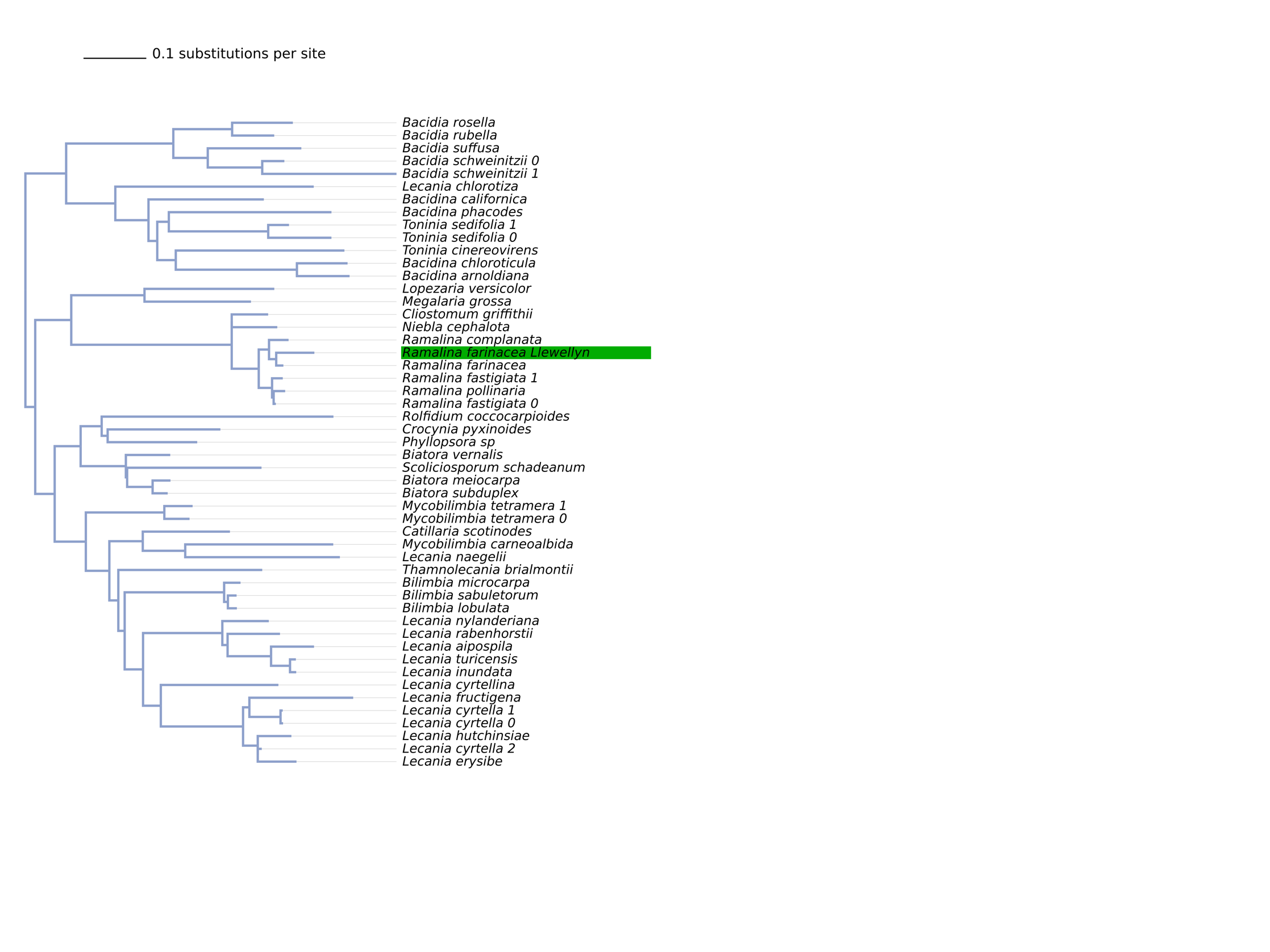


**Supplementary Figure S1:** Subsection of T-BAS *Lecanoromycetes* v2 reference tree based on four loci combined dataset (nucLSU, mitSSU, RPB1 and RPB2) showing *Ramalinaceae* family with our sample highlighted in green as placed by the Evolutionary Placement Algorithm (EPA).

**References**

Buchfink B, Xie C, Huson DH. 2014. Fast and sensitive protein alignment using DIAMOND. Nat. Methods. 12:59–60. doi: 10.1038/nmeth.3176.

Camacho C et al. 2009. BLAST+: Architecture and applications. BMC Bioinformatics. 10. doi: 10.1186/1471-2105-10-421.

Grigoriev IV et al. 2014. MycoCosm portal: gearing up for 1000 fungal genomes. Nucleic Acids Res. 42:699–704. doi: 10.1093/nar/gkt1183.

Laetsch DR, Blaxter ML. 2017. BlobTools: Interrogation of genome assemblies. F1000Research. 6:1287. doi: 10.12688/f1000research.12232.1.

Li H. 2018. Minimap2: pairwise alignment for nucleotide sequences. Bioinformatics. 34:3094–3100. doi: 10.1093/bioinformatics/bty191.

Li H et al. 2009. The Sequence Alignment/Map format and SAMtools. Bioinformatics. 25:2078–2079. doi: 10.1093/bioinformatics/btp352.

Pryszcz LP, Gabaldón T. 2016. Redundans: An assembly pipeline for highly heterozygous genomes. Nucleic Acids Res. 44:e113. doi: 10.1093/nar/gkw294.

Shen W, Le S, Li Y, Hu F. 2016. SeqKit: A cross-platform and ultrafast toolkit for FASTA/Q file manipulation. PLoS ONE. 11:e‌0163962. doi: 10.1371/journal.pone.0163962.

Vaser R, Sović I, Nagarajan N, Šikić M. 2017. Fast and accurate de novo genome assembly from long uncorrected reads. Genome Res. 27:737–746. doi: 10.1101/gr.214270.116.
