# Supplementary figures and images for: First Whole-Genome Sequence and Flow Cytometry Genome Size Data for the Lichen-Forming Fungus Ramalina farinacea (Ascomycota)
Source: Genome Biol Evol. 2023 May 7;15(5):evad074. doi: 10.1093/gbe/evad074 (PMC10195087; doi:10.1093/gbe/evad074)

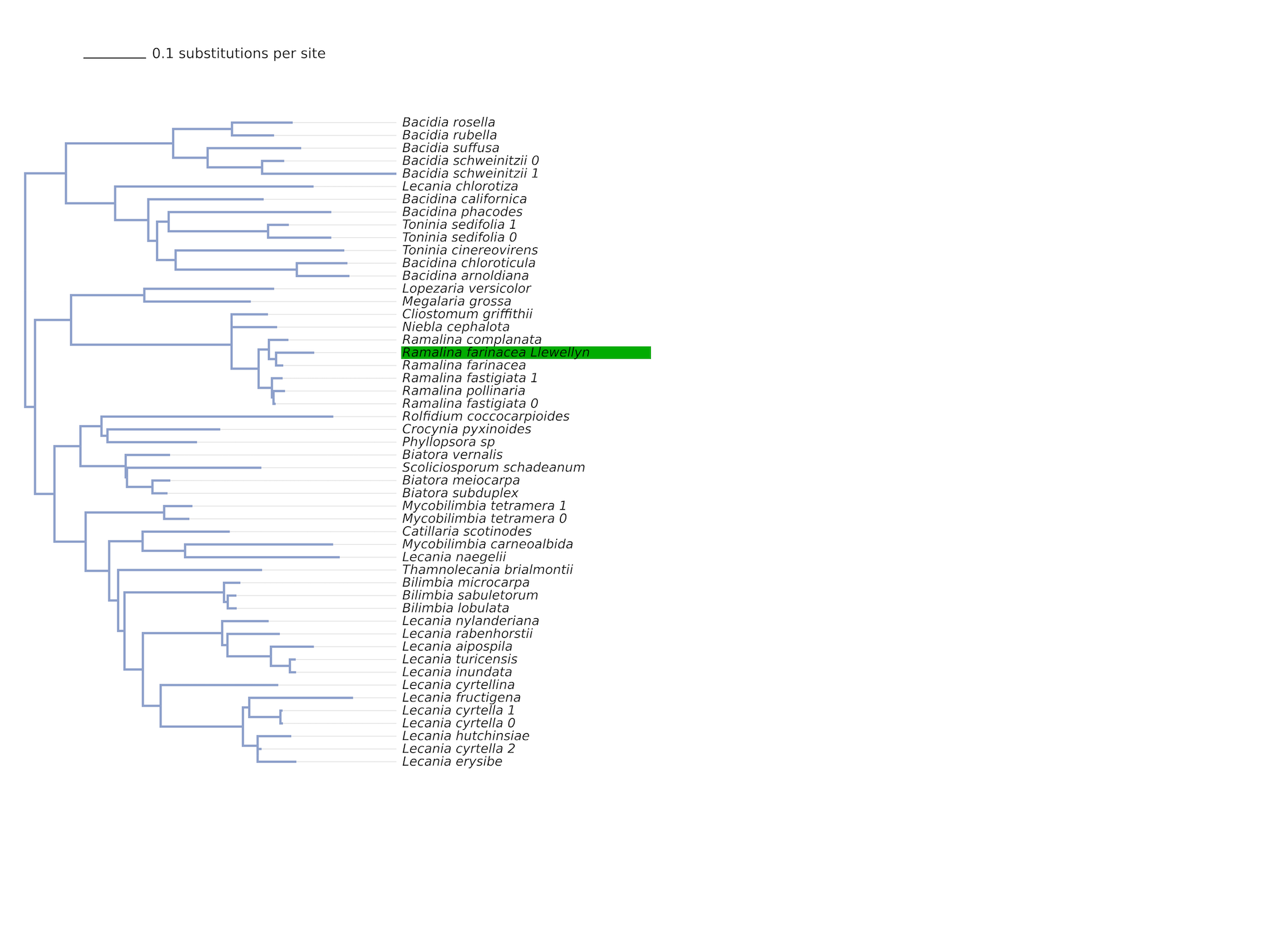

Supplement: evad074_Supplementary_Data [file evad074_supplementary_data.zip › Supplementary_FigureS1.tiff]
